# Lateral Epicondylalgia as a Nociceptive/Nociplastic Pain Condition According to the International Association for the Study of Pain (IASP) Taxonomy: A Theoretical Hypothesis

**DOI:** 10.3390/jcm15218432

**Published:** 2026-10-29

**Authors:** Margarita Cigarán-Mendez, Joshua Cleland, Jorge Rodríguez-Jiménez, Cristina Gómez-Calero, Lars Arendt-Nielsen, César Fernández-de-las-Peñas

**Affiliations:** 1 Department of Psychology, Universidad Rey Juan Carlos, 28922 Madrid, Spain; margarita.cigaran@urjc.es; 2 Department of Rehabilitation Sciences, School of Medicine, Tufts University, Boston, MA 02111, USA; joshua.cleland@tufts.edu; 3 Department Physical Therapy, Occupational Therapy, Rehabilitation, and Physical Medicine, University Rey Juan Carlos, 28922 Madrid, Spain; jorge.rodriguez@urjc.es (J.R.-J.); cristina.gomez@urjc.es (C.G.-C.); 4 Center for Neuroplasticity and Pain (CNAP), Sensory-Motor Interaction Center (SMI), Department of Health Science and Technology, Faculty of Medicine, Aalborg University, DK-9220 Aalborg, Denmark; lan@hst.aau.dk; 5 Department of Gastroenterology & Hepatology, Mech-Sense, Clinical Institute, Aalborg University Hospital, DK-9000 Aalborg, Denmark; 6 Steno Diabetes Center North Denmark, Clinical Institute, Aalborg University Hospital, DK-9000 Aalborg, Denmark

**Keywords:** lateral elbow pain, nociceptive, neuropathic, nociplastic, phenotyping

## Abstract

Lateral epicondylalgia (LE) is a chronic musculoskeletal pain condition characterized by treatment effect heterogeneity. The identification of specific pain phenotypes may help in subgrouping LE patients based on their predominant pain mechanism, thereby supporting the development of more targeted treatments. In the current proposal, we present a conceptual application of the International Association for the Study of Pain (IASP) taxonomy to identify a predominant LE pain phenotype. The clinical presentation of LE is traditionally considered predominantly nociceptive, with the wrist extensor tendon as the main peripheral driver. Thus, the presence of trigger point (TrP)-associated referred pain has also been considered as a potential peripheral nociceptive drive. However, the treatment of peripheral drivers alone has shown limited short-term effects in LE, requiring research beyond possible peripheral inputs. Furthermore, evidence supports the presence of altered pain processing in some individuals with LE, suggestive of nociplastic pain features in a subgroup of patients. The potential presence of nociplastic features would imply the need to address maladaptive neuroplasticity associated with pain processing in these patients with LE. The current proposal hypothesizes that LE is a heterogeneous pain condition in which different pain phenotypes, e.g., nociceptive-alone, nociplastic, or mixed-type, can coexist in some patients with different relevance according to the duration, intensity, stage and severity of the disease. Recognizing the relative predominance of nociceptive and nociplastic phenotypes may guide more individualized treatment selection in individuals with LE. However, these hypotheses require prospective validation before they can be established as a clinical decision-making tool. Future studies should identify high-value care options for the treatment of people with LE, as current approaches only yield short-term effects.

## 1. Introduction

Chronic musculoskeletal pain, excluding spinal disorders, represents the fifth leading cause of years lived with disability (YLD) worldwide [1]. Lateral epicondylalgia (LE) is a chronic musculoskeletal condition affecting 1–3% of the worldwide population [2], with an overall adjusted incidence of 3.4 per 1000 in middle-aged adults [3]. Lateral epicondylalgia has been traditionally considered an overload tendinopathy of the wrist extensors; however, recent advances suggest the presence of predominant peripheral or central mechanisms depending on the specific tendinopathy [4]. In the last decade, there has been an increase in knowledge on the underlying causes and mechanisms of chronic painful tendinopathies; however, several gaps in their pathophysiology are still present. In fact, hypotheses range from considering chronic tendinopathies as primarily inflammatory-driven pathology to a mechanical “failed-healing” model to a complex painful condition where structural changes and inflammatory processes located in the tendon matrix and altered nociceptive pain processing interact at the same time with impairments in motor output [5,6].

The International Association for the Study of Pain (IASP) describes three main pain phenotypes: nociceptive, neuropathic and nociplastic [7]. This classification was initially well received; nevertheless, it has been questioned [8]. First, clinical discrimination between these phenotypes can be difficult as patients may exhibit two phenotypes simultaneously (e.g., mixed-type phenotype), since phenotypes are not exclusive [9]. Thus, clinical presentation of a patient can evolve with time from one predominant phenotype to another. Second, no standardized tools exist for the clinical identification of altered central pain processing in humans [10]. In fact, IASP taxonomy has not been previously applied to painful tendinopathies such as LE.

In 2021, the IASP proposed different criteria to guide the clinical identification of the proposed phenotypes [11]. These criteria are detailed, well designed, and have the potential to be applied in clinical practice [11]. Identifying the predominant pain phenotype could enhance precision pain medicine as, presumably, individuals with chronic pain are likely to respond best to interventions that specifically target the predominant phenotype [12]. Differentiating between phenotypes could be relevant because nociplastic pain may be more challenging to treat than other pain phenotypes; however, this hypothesis has yet to be tested. This paper describes a theoretical framework for clinically identifying a predominant phenotype in individuals with LE by adapting IASP clinical criteria [11].

## 2. Lateral Epicondylalgia as Nociceptive Pain Condition

Nociceptive pain can be defined as “pain attributable to the activation of the peripheral receptive terminals of primary afferent neurons, particularly from non-neural tissues, in response to different noxious stimuli, and clinically, the pain response is proportional to the nociceptive input” [13]. The clinical presentation of typical LE is compatible with a predominant nociceptive phenotype with a potential main peripheral musculoskeletal driver such as the wrist extensor tendon. Evidence supports the presence of increased levels of neuropeptides (e.g., substance P and calcitonin gene-related peptide), inflammatory cytokines (e.g., interleukin 1α and tumor necrosis factor-α) and glutamate receptors at the tendon of the extensor carpi radialis brevis (ECRB) muscle in individuals with LE when compared with pain-free people [14,15]. Furthermore, LE is also commonly associated with degenerative structural changes at the wrist extensor tendon [16,17]. Based on current findings, it seems that the tendon of the wrist extensors could act as a peripheral nociceptive driver in LE; however, pain symptoms are not always associated with tendon structural changes since pain-free individuals can exhibit structural tendon changes, and symptoms can be present without structural tendon changes [18]. In addition, structural tendon changes have not been correlated with other quantitative sensory testing in LE [19,20].

Another hypothesis is that not only the tendon but also the muscle could act as a peripheral nociceptive driver in LE. Muscle pain can activate peripheral nociceptors through the release of inflammatory mediators, e.g., bradykinin, interleukins, serotonin, calcitonin gene-related peptide, substance P, or tumor necrosis factor-α [21]. In an experimental pain model, hypertonic saline injection into the wrist extensors/supinator muscles of healthy people elicited pain referral patterns mimicking sensory and motor symptoms similar to those experienced by patients with LE [22]. Such observations created the idea that LE may also be associated with muscle referred pain, serving as another potential nociceptive source [23].

From a clinical perspective, experimentally induced muscle referred pain can be reproducible using myofascial trigger points (TrPs). Trigger points are defined as “hypersensitive spots located into taut bands of skeletal muscles that are painful upon stimulation, which usually feature referred pain sensations and associated phenomena” [24]. However, the diagnosis of TrPs is based on manual palpation [25] as no quantitative or objective assessment criteria currently exist, leaving uncertainty about the exact nature of their nociceptive source. The presence of muscle TrP-associated referred pain reproducing symptoms of LE has been documented in the literature [26,27]. If we consider TrPs as potential peripheral nociceptive drivers, this could contribute to nociceptive pain features in LE.

From a treatment perspective, in chronic pain conditions with a nociceptive phenotype, management typically focuses on maintaining functional activity and the application of early interventions that target the nociceptive input (driver). Thus, TrP treatment has been found to be effective (low–moderate-quality evidence) for improving pain and function in the short term in individuals with LE [28,29]. Similarly, other therapeutic approaches, such as manual therapy and exercise, targeting peripheral nociceptive drivers such as the tendon have also shown short- or mid-term effectiveness for LE [30].

Based on current data, there is limited evidence that considers LE purely as a nociceptive pain condition, since an exact nociceptive driver is not always identifiable. While this lack of evidence does not exclude the possibility of a predominant nociceptive pain phenotype in some patients with LE, it at least suggests the need to consider other pain phenotypes beyond peripheral nociceptive pain in individuals with LE.

## 3. Lateral Epicondylalgia as Neuropathic Pain Condition

Neuropathic pain should fulfill the following criteria: (1) the presence of a lesion or disease affecting the somatosensory nervous system, whether peripheral or central; (2) symptoms that follow a neuroanatomically plausible distribution corresponding to the affected nervous system; and (3) pain supported by clinical examination findings, as well as laboratory and/or imaging evidence [31].

Typical clinical presentation of LE usually does not meet these criteria for neuropathic pain as no clear evidence of somatosensory nervous system damage has been identified in this population. However, growing evidence suggests that some musculoskeletal pain conditions traditionally not considered as neuropathic, e.g., fibromyalgia or irritable bowel syndrome, may exhibit morphological signs of sensory nervous system pathology such as small nerve fiber pathology [32]. To date, no studies have examined this in individuals with LE [32].

Nevertheless, some models propose that nerve tissue can also be involved in tendinopathy-related pain [33,34]. Interestingly, a recent meta-analysis found that up to 40% of individuals with LE self-reported neuropathic-like symptoms [35]. However, the studies included in this review relied solely on self-reported outcomes commonly used in clinical practice to identify the presence of possible neuropathic-like symptoms, but they did not provide evidence of the presence of nerve damage [36]. Thus, the presence of neuropathic-like symptoms, in the absence of nervous system damage, may also be a manifestation of altered pain processing. In fact, it has been identified that some patients with LE exhibit radial nerve mechanical pain hypersensitivity [37,38]. This finding has led to the application of interventions aiming to decrease neural sensitivity, such as neurodynamic interventions [39].

## 4. Lateral Epicondylalgia as Nociplastic Pain Condition

The term nociplastic pain was defined as “pain that arises from altered nociception despite no clear evidence of actual or threatened tissue damage causing the activation of peripheral nociceptors or evidence for disease or lesion of the somatosensory system causing pain” [40]. Thus, altered central pain processing or central sensitization, defined as an increased responsiveness of the nociceptive neurons in the central nervous system to their normal or subthreshold afferent input by the IASP [41], is considered a potential underlying mechanism of nociplastic pain [7].

Evidence supports the presence of altered pain processing in LE, although definitive conclusions cannot be made at this stage. Data support the presence of bilateral pressure and thermal pain hyperalgesia at the symptomatic area (elbow, low-to-moderate-quality evidence) and bilateral hypersensitivity to pressure pain at a segmental-related area (cervical spine, moderate evidence) suggestive of regional sensitization [42,43,44,45,46,47]. On the contrary, the presence of widespread pressure pain hypersensitivity in distant pain-free areas in patients with LE is inconsistent [44,45,46]. Furthermore, up to 70% of patients with LE experience pain in the cervical/thoracic spine [48], as well as cervical spine impairments in the absence of neck pain symptoms [49]. Another manifestation of regional sensitization is the presence of spreading pain towards the forearm and the dorsal aspect of the wrist, mostly associated with TrP-related referred pain [26,27].

Some studies have found that patients with LE exhibit increased temporal summation and impaired conditioned pain modulation, although these impairments depend on the stimuli applied [50,51]. Furthermore, people with LE also experience altered primary motor cortex organization (greater excitability, inhibition and overlap) [52]. These brain changes in the primary motor cortex may contribute to the presence of bilateral motor deficits, particularly lower reaction time, in people with unilateral elbow tendinopathy [47]. It is possible that some patients with LE exhibit only some features of altered pain processing, and even that subgroups of patients with different degrees of sensitization exist. Jespersen et al. observed that, in patients with LE without signs of tendon inflammation, temporal summation was significantly stronger than in those with ongoing tendon inflammation [53]. In summary, the presence of altered pain processing in individuals with LE seems to be within-subject and stimuli-dependent.

Additionally, fatigue, poor sleep quality and mood disturbances, central nervous system-derived symptoms related to neuro-immune alteration typical of nociplastic conditions [54], have also been observed in individuals with LE [55,56]. The meta-analysis by Mallows et al. identified conflicting evidence regarding the presence of anxiety/depression in LE and found moderate evidence about the presence of catastrophizing and distress in LE [57]. It is important to highlight that referred pain, a hallmark of TrP-associated pain, reflects a manifestation of altered central sensitization influenced by peripheral drivers, as well as increased sympathetic activity and impaired descending pain inhibition [58,59]. In such a scenario, it has been hypothesized that TrPs may act as a long-lasting/persistent peripheral nociceptive input contributing to altered pain processing [60]. Current data indicate that LE is a heterogeneous musculoskeletal pain disorder resulting from the dynamic interplay between peripheral and central mechanisms integrated into a contextual/personal environment. At this point, if altered pain processing is present in some individuals with LE, it would suggest nociplastic pain features. Thus, in these patients, both nociceptive and nociplastic phenotypes could coexist with different degrees of predominance. Further research is needed to confirm or refute this hypothesis.

## 5. Algorithm for Pain Phenotyping of Lateral Epicondylalgia

This section will apply the IASP criteria/clinical reasoning process to identify the predominant phenotype in individuals with LE [11]. Clinicians should recognize that a single patient may meet criteria for two phenotypes (mixed-type) and that a pain phenotype can evolve to another one over time, though no longitudinal study has yet explored this hypothesis. Accordingly, the primary objective should be to determine the predominant phenotype in a particular patient at a specific moment. Thus, this is a continuous process where all steps should be explored in a patient, even though a predominant pain phenotype, e.g., nociceptive or neuropathic, has been initially identified.

### 5.1. Step 1—Pain Duration

Firstly, pain is considered chronic when symptoms are present for at least 3 months [11]. Although LE is a self-limiting condition that can resolve on its own within 6 to 24 months [61], several patients experience pain for years.

### 5.2. Step 2—Pain Distribution/Location

The pain of nociceptive features is usually localized, and it is exacerbated by specific triggers. The pain of neuropathic features should follow the anatomical path of a peripheral nerve. The pain of nociplastic features is generalized and/or widespread [11]. Individuals with typical LE commonly report pain centered around the elbow area, supporting a nociceptive phenotype. Nevertheless, some patients also describe pain spreading towards the forearm and the dorsal aspect of the wrist, mostly associated with TrP-related referred pain [26,27]. This pain pattern does not follow the anatomical trajectory of a peripheral nerve, so it would not be a neuropathic phenotype. Additionally, some individuals with LE also report symptoms in the cervical spine [48,49]. Thus, careful assessment of pain distribution during clinical evaluation is crucial to detect symptom changes from localized (elbow) to regional (forearm, cervical spine) patterns. Using standardized pain drawings is a reliable and valid method to optimize the assessment of a patient’s pain distribution and location [62].

### 5.3. Step 3—Can Symptoms Be Explained by Nociceptive Pain?

The next step is to evaluate whether the pain pattern can be fully explained by nociceptive mechanisms [11]. Based on clinical presentation and the presence of tendon/muscle impairments, typical LE can be firstly considered a nociceptive pain condition. If TrPs are accepted as a nociceptive condition [63], this assumption gains further support. Still, this clinical interpretation should always be interpreted considering the clinical picture of each single patient. For instance, TrP-elicited elbow/forearm pain can only be considered a sign of nociceptive pain without the presence of generalized/widespread pain, and without other signs of central nervous system hypersensitivity. In fact, the identification of symptoms of a nociceptive source of pain does not exclude the presence of other potential phenotypes, so the examination of a patient with chronic pain should include all steps.

### 5.4. Step 4—Can Symptoms Be Explained by Neuropathic Pain?

The typical presentation of LE usually does not fulfill the criteria for neuropathic pain since no evidence of somatosensory nervous system damage has been observed in this population [11]. Nevertheless, the presence of self-reported neuropathic-like symptomatology is possible in this population [39].

### 5.5. Step 5—Identification of Pain Hypersensitivity Responses

The next step involves the identification of hypersensitivity responses associated with central nervous system responsiveness, i.e., hyperalgesic or allodynic responses [11]. Nociceptive pain is mainly characterized by hypersensitivity around the painful area (localized or regional sensitivity), whereas nociplastic pain is commonly characterized by hypersensitivity at the painful area and other potential distant areas (widespread pain sensitivity), although this is not mandatory. It has been found that patients with LE exhibit pressure pain hyperalgesia within symptomatic (localized hyperalgesia) and segmental-related (regional hyperalgesia) areas, but the presence of distant pain-free areas (widespread hyperalgesia) is inconsistent [44,45,46].

### 5.6. Step 6—History of Self-Reported Pain Hypersensitivity

Step 6 consists of the presence of self-reported symptoms of hypersensitivity, particularly allodynia. The presence of cutaneous allodynia in LE has not been specifically investigated. Some studies have used patient reported outcome measures (PROM) for evaluating the presence of neuropathic-like symptoms, including allodynia [35], but no specific study has focused on this type of response in this pain population.

### 5.7. Step 7—Presence of Comorbid Symptomatology

The final step consists of identifying for the presence of (1) hypersensitivity to different stimuli such as sound (phonophobia) or light (photophobia); (2) medical conditions; (3) other nervous system-associated symptoms including sleep quality, fatigue and cognitive disturbances [11]. First, sensitivity to chemical stimuli is not commonly experienced by patients with LE. Second, some patients with LE can report comorbid medical conditions of nociplastic features such as fibromyalgia [64,65]. There is a small study reporting the presence of fibromyalgia syndrome in 40% of subjects with LE [66]. No population-based studies investigating the prevalence of fibromyalgia or other chronic medical conditions in individuals with LE have been published. Third, the presence of generalized symptoms such as fatigue, mood disorders or sleep disturbances can be present in individuals with LE [55,56], but they are not extensively common as in other pain conditions.

**Figure 1:**
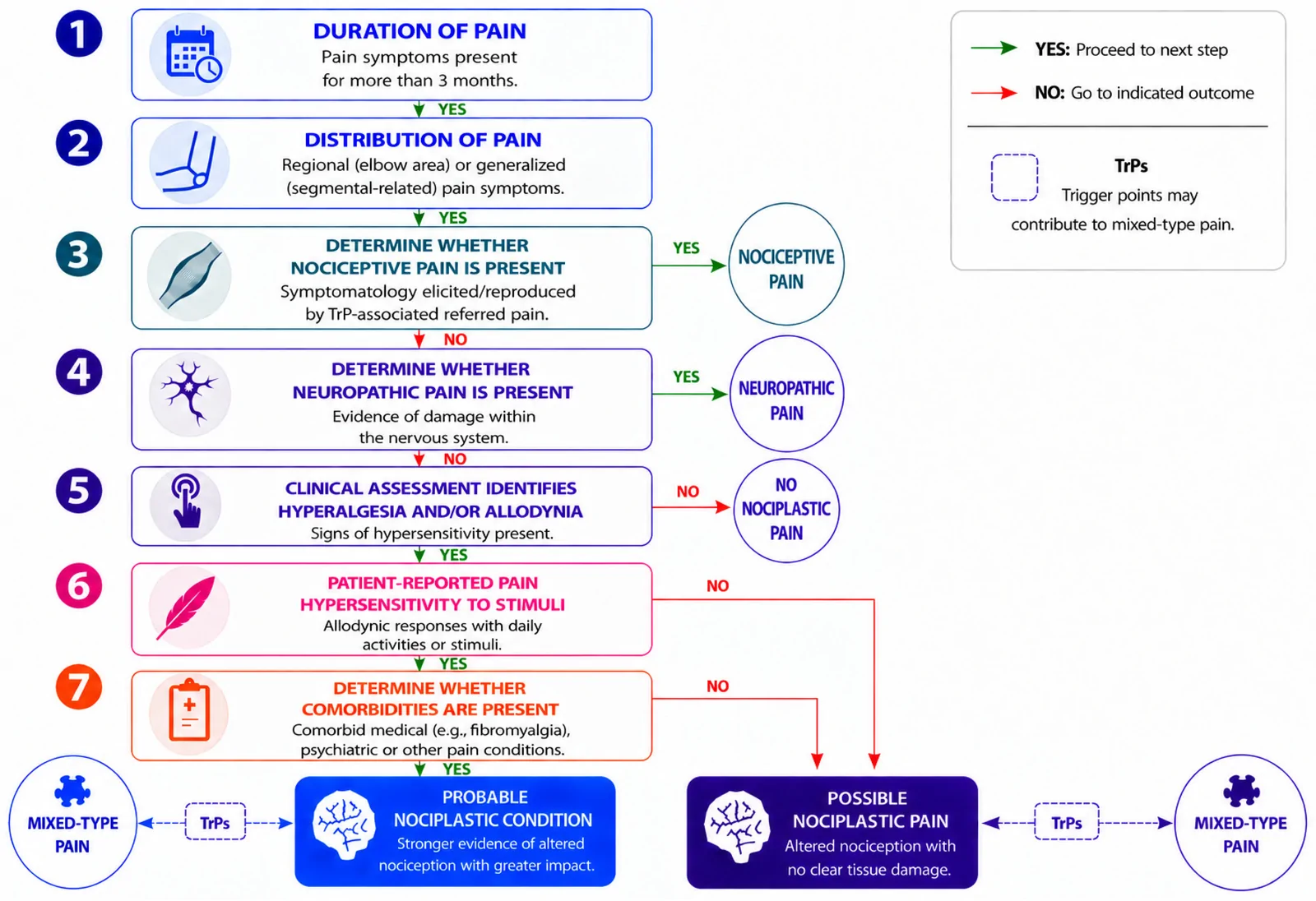
Proposed clinical decision-making tree for identifying the pain phenotype of patients with lateral epicondylalgia (LE). Adapted from 2021 IASP clinical criteria [11].

If the patient’s presentation meets all criteria, the classification of “probable nociplastic pain” should be adopted [11]. Figure 1 presents a conceptual framework algorithm, not yet validated, based on the IASP criteria adapted for identifying the predominant pain phenotype in people with LE. Although initial reliability testing using clinical vignettes for some musculoskeletal pain conditions has shown promising results [67], the reliability and validity of this algorithm for specific pain conditions has not been investigated [11]. In particular, no study has examined the reliability and validity of these criteria in individuals with LE. Finally, future clinical trials evaluating the prognostic value and responsiveness of this diagnostic algorithm for the improvement of treatment outcomes are needed.

## 6. Considering Pain Phenotype in Lateral Epicondylalgia Treatment

The current paper proposes that, given their heterogenous clinical presentations, patients with LE should be treated based on their underlying pain phenotype rather than using a one-size-fits-all approach. In fact, clinical guidelines for managing people with LE are hindered by heterogeneous interventions and the condition’s prognosis, ranging from quick resolution of the symptoms following simple advice in some subjects with a good or excellent prognosis to patients with long-lasting problems regardless of treatment [68]. It could be hypothesized that the extent of nociplastic features in patients with chronic pain conditions, including LE, could be inversely related to the effectiveness of treatments targeting only peripheral mechanisms. No study has yet investigated whether phenotype-matched interventions improve clinical outcomes in this population. The current proposal highlights the relevance of identifying individuals with nociplastic pain features in guiding the selection of the most appropriate therapeutic strategies.

There is no consensus regarding the most efficacious treatment for people with LE [69,70,71,72]. Numerous conservative treatment options exist, but outcomes associated with each intervention differ depending on the clinical presentation of the patient [68,69,70,71,72]. This could very well be related to the specific type of pain features a patient with LE presents with on the initial examination [73,74]. The overwhelming majority of treatments for LE focus on tendon pathology [70,71]. Current evidence has shown that exercise programs that include tendon loading are effective for managing people with LE [61]. Other treatments with considerably less evidence that are also applied for LE include lifestyle habit modification, manual therapy, taping, bracing, extracorporeal shockwave therapy, corticosteroid injection, autologous blood injection or surgery [70]. Nevertheless, no single intervention has consistently demonstrated long-term effect across all patient populations, perhaps due to the heterogenous nature of the condition.

It appears that some people present with a solely nociceptive clinical presentation, which is most frequently associated with localized tendon pain. Conversely, some patients may potentially present with pain characteristics resembling a mixed nociceptive–nociplastic phenotype due to altered nociception and pain processing. Therefore, identification of the specific pain phenotype may result in improved management of the condition.

The discrepancy observed in patient responses to various treatments might be partly attributed to the fact most treatment strategies specifically target tissue pathology in the periphery. This treatment approach assumes that pain generation is primarily associated with peripheral nociception. However, growing evidence suggests that some individuals with LE have several signs of altered pain processing suggestive of a potential nociplastic phenotype. For these patients, we can hypothesize that treatments solely targeting peripheral drivers could potentially be insufficient due to the presence of altered pain processing responsible for their ongoing symptoms. Hence, clinical identification of predominant peripheral or central drivers is important [75]. Utilizing the IASP’s clinical criteria may be beneficial for helping the clinician to recognize patients who suffer from a mixed presentation of both a local disorder and a pain modulating process [11], although no study has investigated this yet. In the process of developing a treatment program for patients with LE, clinicians would need to determine whether nociplastic elements are involved and the extent to which these features influence clinical presentation. Acquiring this knowledge might assist in answering the question of why subjects experience different responses to various treatments despite having a similar condition.

Growing evidence suggests that ongoing nociceptive input from peripheral drivers (e.g., tendons, muscles joints) may facilitate sensitization [12]. Considering this, patients who initially present with a predominantly nociceptive profile may experience the gradual development of features suggestive of a mixed nociceptive–nociplastic phenotype. This progression may occur in those subjects with long-standing symptoms, widespread pain, increased pain sensitivity, or clinical disproportionate responses to the extent of the actual tissue damage.

The current conceptual proposal suggests that clinicians’ ability to recognize differences in phenotypes could have important implications for patient treatment and individual outcomes. Patients presenting with symptoms that are primarily nociceptive in nature might respond best to interventions aimed at addressing the local pathology, such as TrP management, progressive exercise programs, modification of daily activities, and approaches designed to specifically focus on improving function. In comparison, patients displaying more nociplastic features can potentially benefit from a broader, multidimensional management approach that focuses beyond the peripheral drivers. This proposal is highly relevant since it might be likely that tendon/muscle pathology may serve as the initial driver of nociceptive input, yet long-term functional outcomes might also depend on addressing any maladaptive changes within the central nervous system in the presence of altered nociceptive processing. Consequently, the management of a predominant nociceptive appearance may rely primarily on peripheral driver tissue-directed (“bottom-up”) approaches, whereas individuals with mixed nociceptive–nociplastic phenotypes need to extend beyond the peripheral drivers to a “top-down” treatment strategy focusing on pain modulation, behavioral influences, and broader nervous system involvement. In such a scenario, our proposal would be that treatment programs should include interventions intended to improve dysregulated pain, such as pain neuroscience education, graded exposure to movement and activity, or cognitive–behavioral strategies. For instance, exercise programs have been shown to be effective in individuals with LE; however, no type of exercise has shown superiority against other interventions [76]. It has been proposed that underlying pain phenotypes must be considered to optimize exercise prescription, mostly in individuals with a nociplastic phenotype [77]. However, despite the constantly growing evidence supporting the concept of directing treatment toward underlying pain mechanisms in a variety of primary musculoskeletal disorders, there remains a paucity in the literature regarding studies examining whether pain phenotype management approaches enhance long-term outcomes in patients with LE. Future studies should examine if applying the IASP criteria can assist clinicians in determining the most appropriate intervention strategy in individuals identified as being at risk for poorer outcomes and improving overall treatment effectiveness for individuals with persistent LE.

## 7. Conclusions

Lateral epicondylalgia has primarily been considered a tendinopathy in which the pain generator seems to be primarily nociceptive, arising from pathology of the common extensors. However, evidence indicates that the clinical presentation of LE is far from being homogeneous. Many subjects, particularly those with typical LE, exhibit features consistent with a primary nociceptive phenotype, but, in some studies, other individuals have exhibited signs of altered pain processing (e.g., widespread hyperalgesia, enhanced temporal summation or altered endogenous pain modulation), suggesting nociplastic features. Based on the current data, we hypothesize that a subset of patients with LE may exist with a potential mixed nociceptive–nociplastic phenotype. This paper presents a clinical decision-making tree to help in the classification and phenotyping of individuals with LE. It is currently unknown whether recognizing the coexistence of nociceptive and nociplastic features will lead to new tools for developing precision pain management strategies and improving treatment options for individuals with LE. Future clinical trials should explore the hypotheses presented in this paper.

## Data Availability

No new data were created or analyzed in this study. Data sharing is not applicable to this article.
